# Size-Independent Nucleation and Growth Model of Potassium Sulfate from Supersaturated Solution Produced by Stirred Crystallization

**DOI:** 10.3390/molecules29010141

**Published:** 2023-12-26

**Authors:** Yayuan Zheng

**Affiliations:** Department of Chemical Engineering, Sichuan University of Science & Engineering, Zigong 643000, China; zhengdan0830@163.com

**Keywords:** potassium sulfate, nucleation, growth, stirred crystallization, population balance equation

## Abstract

This paper explores the kinetics of the crystallization of potassium sulfate in a stirred bed crystallizer through experimental investigation. Employing classical nucleation theory, the homogeneous and heterogeneous nucleation mechanisms of potassium sulfate were investigated. The induction time and critical nucleation parameters, including the surface tension (*γ*), critical nucleation radius (r*), critical nucleation free energy (ΔG*), and critical nucleation molecule number (i*), were meticulously determined under varying temperatures and supersaturation ratios. The experimental findings revealed that as the temperature and supersaturation ratio increased, the induction time, critical nucleation free energy, critical nucleation radius, and critical molecule number decreased whereas the nucleation rate increased. The crystalline shape remains relatively unaltered with respect to temperature and supersaturation ratio, yet the particle size (D_10_, D_50_, D_90_) increases as the supersaturation and temperature increase. The variations in the measured nucleation parameters align well with the predictions of classical nucleation theory. Furthermore, the kinetic equations of crystal nucleation and the growth rate in a stirred crystallization system were fitted using population balance equations. The results demonstrate that the growth rate increases with increasing supersaturation and stirring rates. Additionally, the effects of the parameters in the nucleation rate equation suggested that the suspension density exerted the greatest influence, followed by the supersaturation ratio and stirring rate. This extensive research provides invaluable theoretical guidance for optimizing the crystallization process and designing industrial crystallizers.

## 1. Introduction

Potassium sulfate serves is a fundamental raw material for the synthesis of various potassium salts, including potassium carbonate and potassium persulfate [1]. Potassium sulfate is extensively employed in various industrial sectors, including glass production, dye production, fragrance manufacturing, the pharmaceutical industry, and more. Its distinctive features include low moisture absorption, resistance to agglomeration, and an excellent physical state, rendering it an easy–to–apply water–soluble potassium fertilizer. Additionally, it possesses physiological acidity, making it an indispensable chemical fertilizer [2,3]. At present, the prevailing method for producing potassium salts is through the evaporation crystallization technique [4,5]. However, this method has several drawbacks, such as high operating temperatures that incur substantial energy consumption, high investment costs associated with expensive equipment, and poor quality of the recovered salts resulting from uncontrolled supersaturation [6]. Consequently, the implementation of low–energy desalination technology that effectively controls the degree of supersaturation is highly desirable.

The crystallization of potassium sulfate from aqueous solution has been the subject of numerous studies. Presently, the advancement of precise simulations that delineate particle evolution patterns is pivotal for optimizing process efficiencies and manipulating particle dimensions and distributions. Zheng meticulously explored the influence of fragmented particles on the particulate dynamics and the subsequent evolution of the size distribution within a spray fluidized–bed crystallizer [7]. Kobari revealed that figures for the quantity density of formed primary nuclei and the count of total crystals (totaling both formed primary nuclei and mature secondary nuclei) were derived as elements of our time calculation process [8]. L. Nemdili explored the solubility, metastable zone width (MSZW), induction time, and crystallization kinetics of potassium sulphate–water [9]. Tseng experimentally observed that secondary nucleation during batch crystallization can be suppressed by using adequate seed loading [10].

The process of supersaturation process is predominantly influenced by the growth of crystals, which involves mass, momentum, or thermal transport through the flowing fluid. The primary objective of agitated crystallization is to produce products characterized by optimal dispersion, uniform particle size, and homogenous morphology. Consequently, it is imperative to thoroughly investigate the nucleation and growth kinetics of agitated crystallization to understand the influence of the initiation process on the kinetics of crystal nucleation and growth. The field of wastewater desalination has witnessed an increasing focus on stirring crystallization due to its potential to yield products exhibiting favorable dispersion, uniform crystal size, and uniform morphology. Consequently, a detailed examination of the stirring crystallization kinetics is imperative. At present, few studies have examined nucleation parameters during potassium salt crystallization.

In this paper, the induction period of potassium sulfate was measured experimentally. Based on the principles of classical nucleation theory, the nucleation parameters pertaining to the supersaturation ratio and temperature were derived. Additionally, the effects of temperature and supersaturation on K_2_SO_4_ crystal morphology and particle size were elucidated. The influence of crystal nucleation and growth kinetics on the crystallization process can be elucidated by analyzing the underlying mechanisms of nucleation and effectively manipulating the nucleation pathways. Consequently, the process can be optimized to provide theoretical guidance for effective control.

## 2. Result and Discussion

### 2.1. Primary Nucleation Kinetics

#### 2.1.1. Crystal Morphology

**Figure 1:**
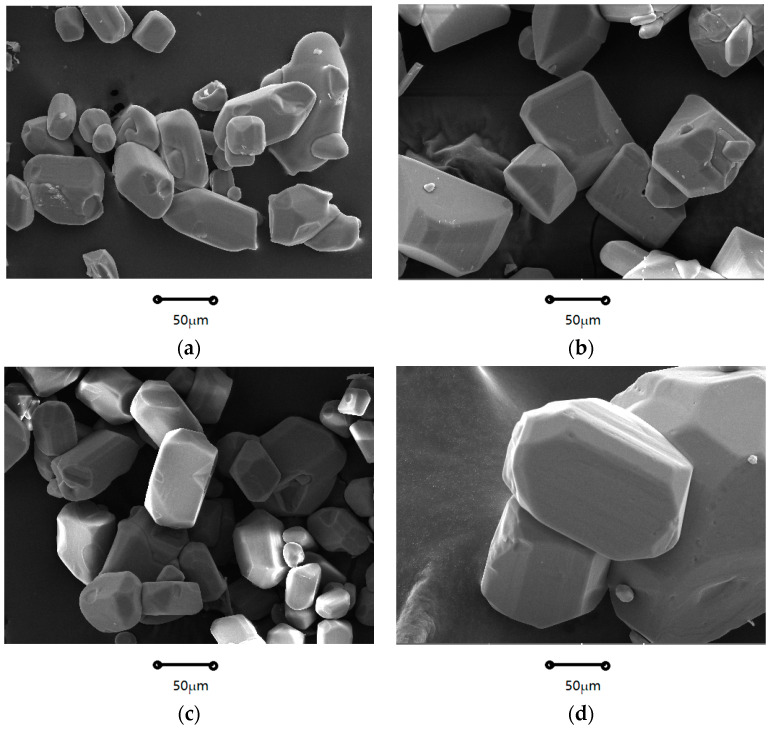
SEM images of K_2_SO_4_ crystallized from different supersaturation at 328 K (**a**) 1.20; (**b**) 1.22; (**c**) 1.25; (**d**) 1.28.

**Figure 2:**
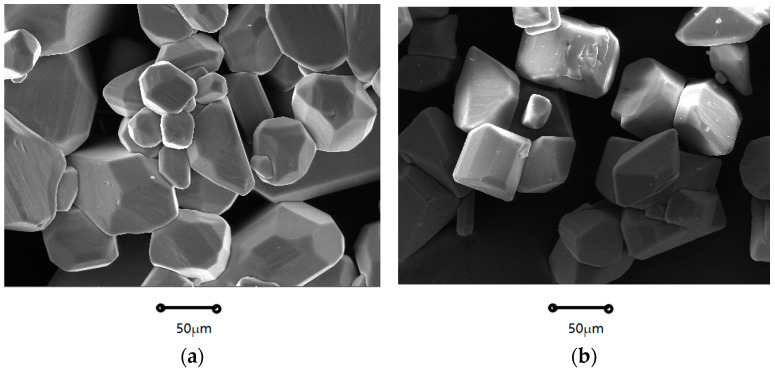

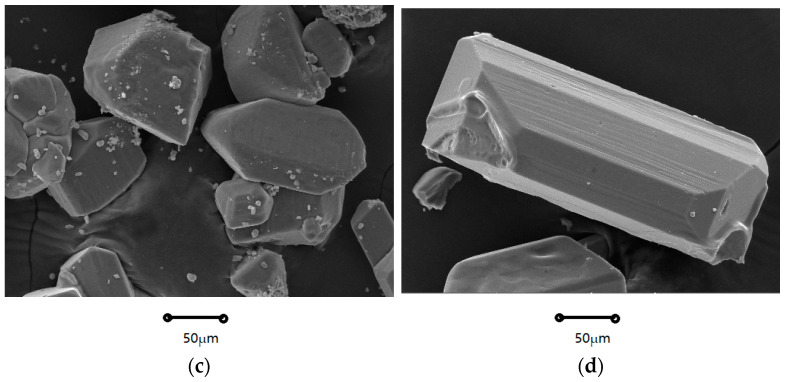
SEM of K_2_SO_4_ crystallized from different supersaturation ratios at 328.15 K (**a**) 1.20; (**b**) 1.22; (**c**) 1.25; (**d**) 1.28.

**Figure 3:**
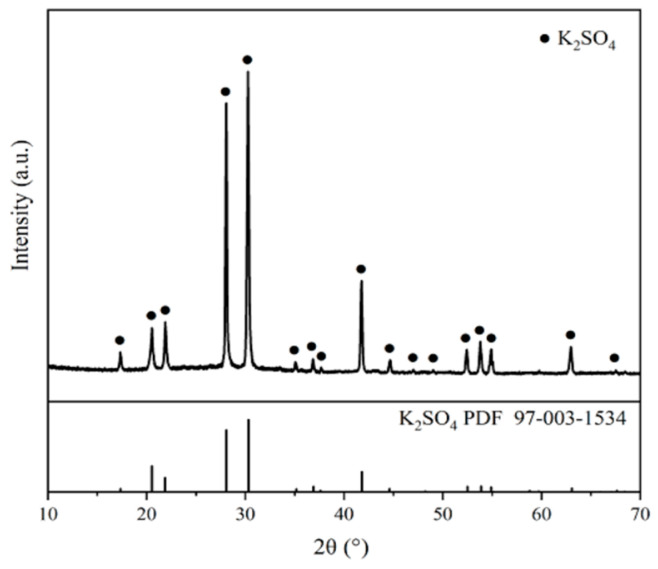
The experimental XRD patterns of K_2_SO_4_. (T: 328 K; S: 1.20).

**Figure 4:**
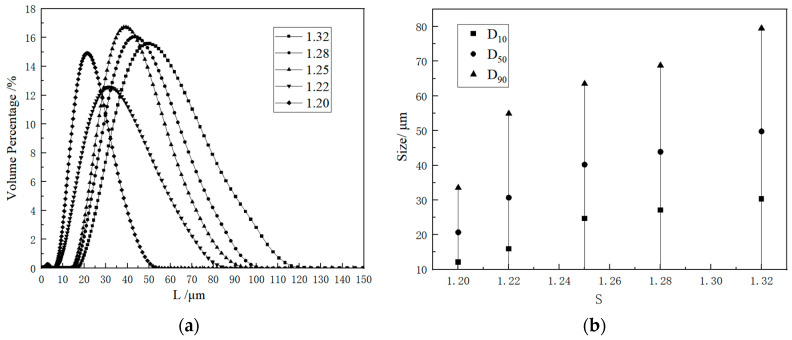
(**a**) Effects of supersaturation on CSD. (**b**) Particle size changes with varying supersaturation at 328 K.

**Figure 5:**
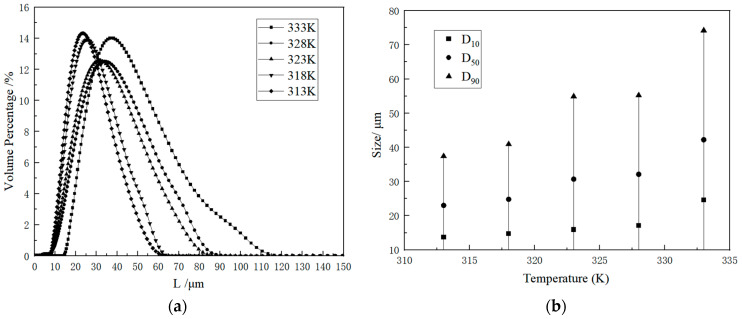
(**a**) Effects of temperature on CSD. (**b**) Particle size changes with varying temperatures at S = 1.2.

As Shown in Figure 1 and Figure 2, the crystal morphologies demonstrated remarkable uniformity, notable regularity, and classical rectangles with smooth surfaces and distinct edges. The crystal morphology predominantly displays a classical cuboid shape. The crystalline product was characterized by X-ray diffraction, and it can be observed from Figure 3 that the system consisted solely of K_2_SO_4_ in an anhydrous state. Notably, as the supersaturation ratio and temperature progressively increased, the particle size of the precipitated crystals gradually increased, as depicted in Figure 4 and Figure 5. When fixing the crystallization temperature, it became evident that the particle size (D_10_, D_50_, and D_90_) increased in tandem with the escalation of the supersaturation ratio. Conversely, when the supersaturation remained constant, the average particle size increased with an increase in the crystallization temperature. A comprehensive analysis of the particle size revealed that the crystals obtained through suction filtration possess an average size ranging from 10–120 μm, exhibiting an unimodal distribution.

It is apparent that the crystals obtained through suction filtration must have undergone a growth period. While the crystallization temperature has the capacity to elevate the nucleation rate, it exerts a more profound influence on the crystal growth process, leading to the production of larger particle sizes. From the data obtained through the particle size analysis, it can be inferred that at higher saturation, crystal growth is favored, and consequently, the crystal particle size increases. Consequently, both elevated crystallization temperatures and low saturation serve as conducive conditions for increasing crystal particle size. 

#### 2.1.2. Nucleation Mechanism

**Figure 6:**
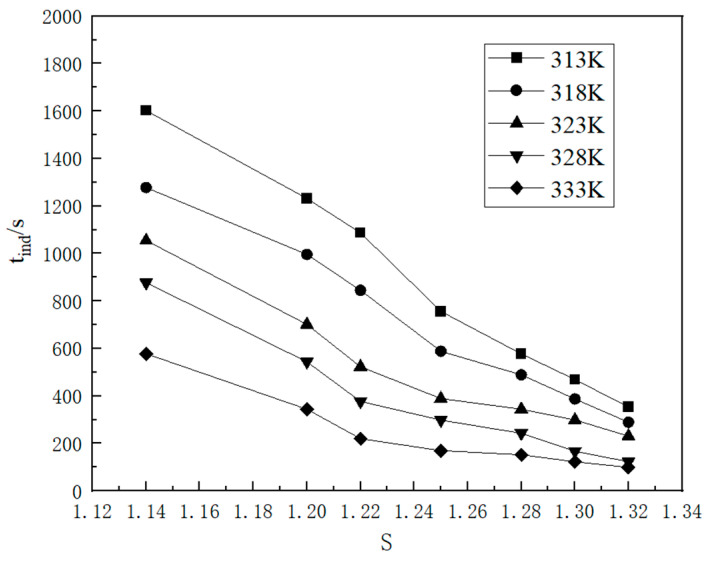
The relationship between the supersaturation ratio and induction period at various temperatures.

The nucleation induction time of potassium sulfate crystals exhibits a specific relationship with the supersaturation ratio and temperature, as shown in Figure 6. Remarkably, the induction time exhibited a nonlinear decrease as the supersaturation ratio and temperature were increased. According to the principles of classical nucleation theory, it is evident that a lower nucleation induction time corresponds to a higher primary nucleation rate, thereby highlighting the significant influence of both the supersaturation ratio and temperature on the crystal nucleation process. Under different conditions, the dominant factor governing the nucleation process was altered. When the supersaturation ratio is high, the concentration of the solute in the solution is elevated, thereby augmenting the probability of solute molecules collision, thereby facilitating nucleation, and reducing the induction time. Conversely, as the supersaturation ratio gradually increased and reached the high supersaturation region, the impact of solute molecule collisions on the induction time gradually diminished, resulting in a flatter curve. When the supersaturation ratio decreases with increasing nucleation temperature, the supersaturation ratio it plays a dominant role in the nucleation process, leading to a shorter time required to initiate nucleation. Conversely, when the supersaturation ratio increases with decreasing nucleation temperature, temperature plays a pivotal role in accelerating the nucleation process. Although the supersaturation ratio serves as the driving force for the crystallization process, it does not always predominate. Both the supersaturation ratio and the nucleation temperature play pivotal roles in regulating the crystallization process.

**Figure 7:**
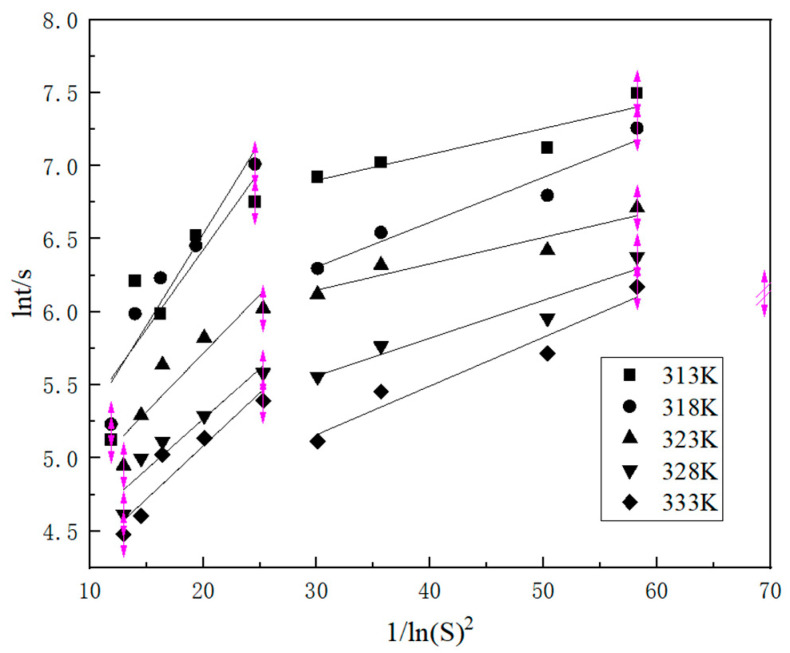
The plots of ln($t_{ind}$) versus 1/ln^2^*S*.

The relationship between homogenous and heterogeneous nucleation in the case of K_2_SO_4_ is illustrated in Figure 7. A linear correlation was observed between the induction time and the supersaturation ratio. The two lines, differing in their slopes, represent the distinct nucleation mechanisms of homogenous and heterogeneous nucleation, respectively. Under high supersaturation conditions, the influence of homogenous nucleation becomes more pronounced. When the supersaturation ratio, S, exceeds 1.25, homogenous nucleation takes the lead. Conversely, when S was less than 1.22, heterogeneous nucleation became the dominant mechanism. When the range between 1.22 and 1.25 is surpassed, the two nucleation mechanisms engage in a competitive interplay, giving rise to a state of transitional nucleation mechanism.

#### 2.1.3. Nucleation Parameters

**Figure 8:**
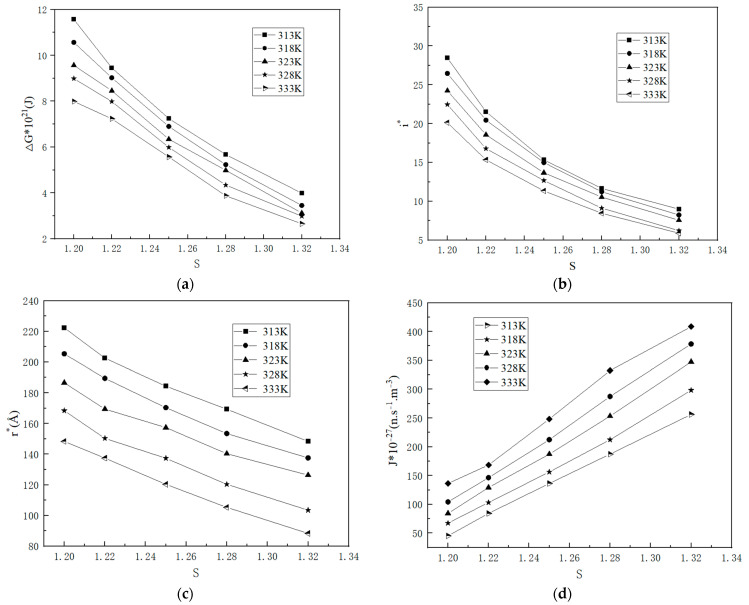
The relationship between the supersaturation ratio and nucleation parameters at various temperatures (**a**) critical nucleation free energy; (**b**) the number of formula units in the critical nucleus; (**c**) the radius of the critical nucleus; (**d**) nucleation rate.

Based on the analysis of Figure 8, it is evident that the classical nucleation theory enables the determination of the nucleation parameters. As the temperature and supersaturation ratio increase, the critical nucleation free energy ΔG*, the critical nucleation radius r*, and the critical nucleus size i* decrease while the nucleation rate J* increases. These experimental findings align well with the classical nucleation theory, thereby providing further verification that both the supersaturation ratio and temperature play a significant role in governing the nucleation process. Consequently, at a specific temperature, an increase in the supersaturation ratio can effectively enhance the nucleation rate and reduce the induction time. Compared to temperature, the impact of the supersaturation ratio on nucleation is considerably more profound.

**Figure 9:**
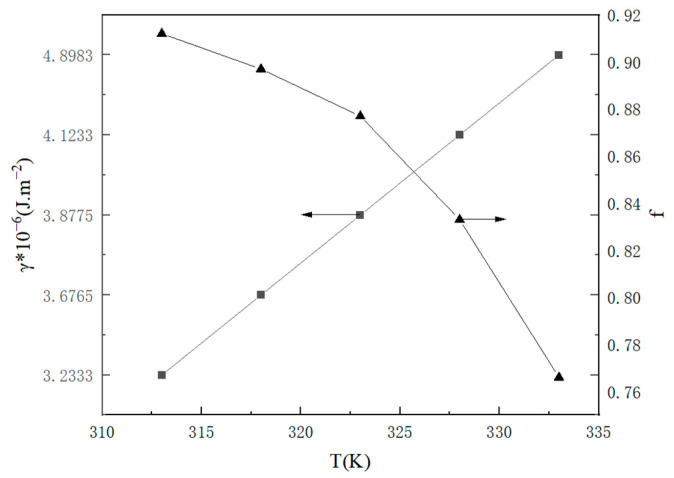
The *γ* and *f* for homogeneous nucleation of K_2_SO_4_.

As shown in the data in Figure 9, the surface entropy factor values for the nucleation and crystallization of potassium sulfate were consistently less than 3. Consequently, it can be hypothesized that potassium sulfate undergoes crystallization via a continuous growth mechanism within this particular system.

### 2.2. Stirring Crystallization Kinetics

#### 2.2.1. Kinetic Data

**Figure 10:**
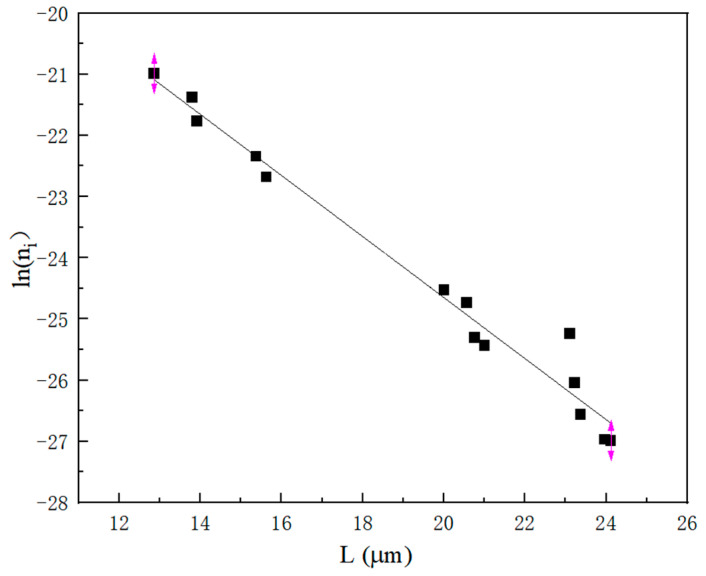
The relationship between crystal particle number density and particle size.

According to Figure 10, a linear relationship is evident between the number density of the crystals and the size of the particles. Consequently, it can be assumed that the rate of crystal growth is independent of the particle size. This study has opted for a growth model that was independent of particle size, and the kinetic parameters were obtained through a nonlinear regression calculation.

#### 2.2.2. Influence Factor

**Figure 11:**
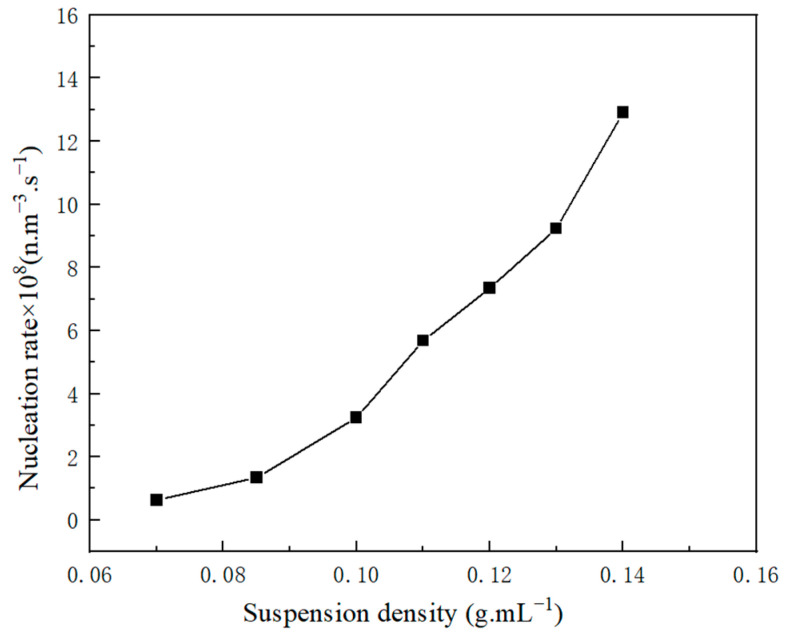
The relationship between nucleation rate and suspension density.

**Figure 12:**
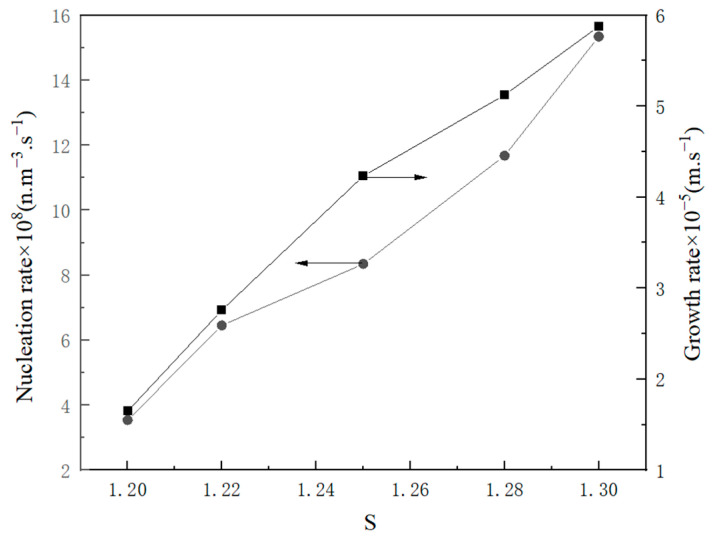
The relationship between nucleation, growth rate, and supersaturation ratio.

The impact of the suspension density on the rate of crystal nucleation is depicted in Figure 11, where the rate of crystal nucleation increases with the increasing suspension density. The presence of suspended particles in the solution can stimulate the formation of secondary nucleation sites, thereby augmenting the likelihood of particle collisions. Figure 12 shows the effect impact of supersaturation on crystal nucleation and growth. Excessive supersaturation can hinder crystal growth due to intense competition between the nucleation and growth processes. A high supersaturation level results in excessively high nucleation rates, inadequate growth space, diminutive particle size, uneven distribution, and inferior crystal quality. The optimization of crystal quality can be achieved by selecting an appropriate supersaturation level during industrial crystallization.

**Figure 13:**
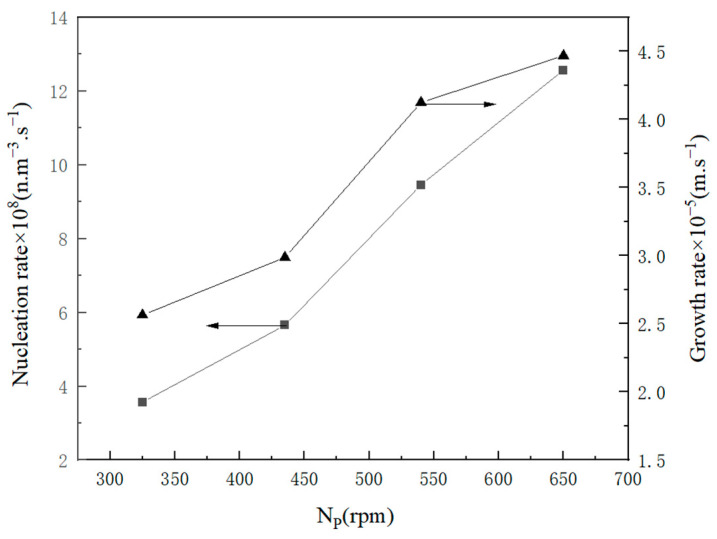
The relationship between crystallization kinetic rate and stirring rate.

Stirring can have a direct impact on the contact process between crystals and crystals, as well as between crystals and container walls (as depicted in Figure 13). When the stirring rate was increased, the kinetic rate of the crystal growth also increased. However, increasing the stirring intensity can result in severe collisions between the solutes and crystal nuclei, leading to prompt spontaneous nucleation, which is unfavorable for crystal growth.

#### 2.2.3. Statistical Analysis of Crystal Parameters

It is indeed beneficial to explore the impact of each variable on the crystallization parameters. The experimental data were fitted to a polynomial equation, and the corresponding coefficients are presented in Table 1. The fit’s quality is indicated by the determination coefficients (*R*^2^).

Table 1 displays that the supersaturation ratio exponents for the nucleation and growth rates are 0.3445 and 0.4655, respectively. It is apparent that the index for the growth rate is higher than that for the nucleation rate, suggesting that as the supersaturation ratio of the system increases, both the nucleation and growth rates of the crystal display a corresponding increase. However, it is important to note that a high supersaturation ratio is not necessarily advantageous for the production of crystals with a significant average size during crystallization. Rather, an elevated supersaturation level provides crystals with enhanced kinetic energy, enabling them to form clusters more rapidly. Consequently, this could lead to an inferior purity and morphology of the final product and may even result in severe scaling within the equipment. Additionally, the supersaturation ratio index of the crystal growth rate derived from the crystallization process stands at 0.7455, suggesting that the crystal growth process may be governed by surface reactions. Notably, all kinetic rates are influenced by the stirring rate, while the crystal growth rate remains independent of the suspension density. As such, the crystal growth rate does not significantly increase with the elevation of suspension density. Taking into account the impact of suspension density on the nucleation rate and growth rate, it becomes evident that a higher suspension density proves to be beneficial for the effective process of crystallization.

**Figure 14:**
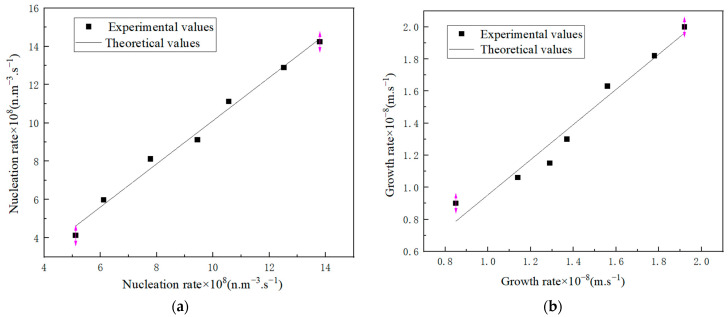
Comparison between experimental and theoretical kinetics rate for K_2_SO_4_ crystallization: (**a**) nucleation rate and (**b**) growth rate.

A comparison between the experimental and calculated rates is depicted in Figure 14, where it can be observed that the mean errors between the calculated and experimental values were 7.65% (a) and 8.23% (b), respectively.

## 3. Material and Methods

### 3.1. Materials

Shanghai Aladdin Biochemical Technology Co., Ltd. (Shanghai, China), a renowned supplier in China, provided a high–purity K_2_SO_4_ solution with a purity of 99.5% or higher and anhydrous ethanol. Deionized water was used to prepare the aqueous solutions. Meticulously selected K_2_SO_4_ crystals, with minute sizes, were placed inside the STC, a state–of–the–art stirred crystallizer. No further purification was performed, demonstrating the exceptional quality of these chemicals.

#### 3.1.1. Experimental Procedure

**Figure 15:**
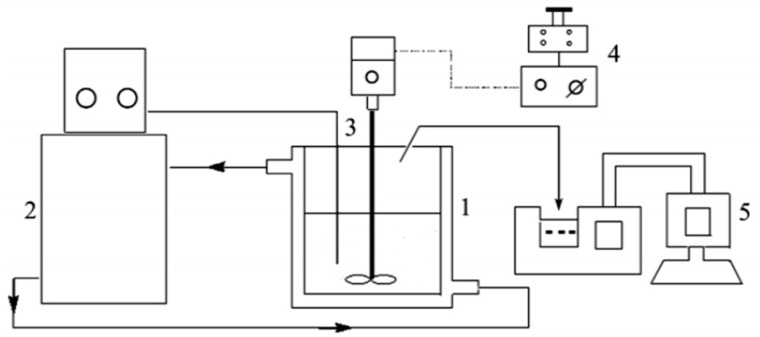
Experimental setup for stirred crystallization. (1) Jacketed crystallizer; (2) Thermostatic circulating water; (3) Thermoelectric couple; (4) Agitator; (5) Laser Particle Size Analyzer.

A schematic of the stirred crystallization process is shown in Figure 15. To begin the experiment, a saturated solution was poured into a crystallizer and heated to a specified temperature. The constant temperature system was activated, and consistent temperature regulation was ensured by adjusting the stirring rate as required. After a significant amount of crystals had precipitated, the system was analyzed by periodic sampling while diligently recording the corresponding time. Subsequently, sampling intervals were initiated every 5 min, and approximately 5 mL of the crystal slurry was extracted. Subsequently, the collected sample was weighed, and the corresponding mass was meticulously recorded. The resulting filter cake was filtered and vacuum–dried. Once dried, the resulting crystal sample was weighed and the corresponding mass was meticulously recorded. Finally, the suspension density of the crystal slurry and the degree of supersaturation in the solution were calculated. The particle size distribution (CSD) of the dried crystal product was determined using a Malvern Mastersizer particle–size analyzer. The crystal size distribution (CSD) of the pieces was measured using a particle analyzer (Malvern Mastersizer Hydro 3000 MU, Malvern Panalytical, Chipping Norton, Australia).

#### 3.1.2. SEM

In this study, a Hitachi S3400N (Hitachi, Tokyo, Japan) scanning electron microscope was used to scan the catalysts. This microscope uses a tungsten filament lamp as its electron source and is equipped with a movable quad–lens column. Before scanning, the catalyst was prepared by adding alcohol and subjecting it to ultrasonic dispersion to ensure uniform mixing. A suitable amount of the catalyst was then removed using a pipette and placed on a sample stage containing a conductive adhesive.

#### 3.1.3. XRD

This paper used a Brucker D8 X-ray diffractometer (Brucker, Elk Grove, IL, USA) to characterize the crystal structures of the seven different catalysts in various proportions. The X-ray diffraction (XRD) was performed using a copper Kα radiation source with a wavelength of 1.5406 Å, operating at 40 kV and 40 mA. The scan speed was set to 2°/min, and the diffraction angle (2θ) ranged from 10° to 70°.

### 3.2. Mathematical Modeling

#### 3.2.1. Population Balance Equations (PBEs)

This paper presents a mathematical model of the crystallization process, namely the Population Balance Model (PBM), which is based on the Numerical Density Function (NDF). This model incorporates mass and energy balance equations to comprehensively elucidate the crystallization kinetics. For intermittent crystallization, the PBM assumes that the crystal growth follows the ΔL rule, thereby suggesting that the growth rate is independent of particle size [11]:

(1) $$\frac{\partial n}{\partial t}+\frac{\partial \left(Gn\right)}{\partial L}+n\frac{d\left(\log V\right)}{dt}+\frac{Q}{V}n=\frac{Q_{i}}{V}n_{i}+\left(B-D\right)$$

The growth rate was expressed as *G*, where *B* and *D* represent the birth and death functions of the crystals, respectively. *Q* represents the outflow rate of the supersaturated solution. The particle number density (*n_i_*) represents the number of crystals within a size range of Δ*L_i_* per unit volume of suspension and can be simplified as follows:

(2) $$n_{i}=\frac{M_{T}V_{i}\%}{k_{v}\rho {\left(\bar{L_{i}}\right)}^{3}\Delta L_{i}}$$

where *M_T_* denotes the suspended density, *V_i_* indicates the crystal volume fraction between sizes *i* − 1 and *i*, *k_v_* is the volume shape factor, and *L_i_* is the average particle size of the *i*th channel. The number of particles present in the ith channel (*N_i_*) can be approximated as follows:

(3) $$N_{i}=n_{i}\times \Delta L$$

Under the assumptions that there is no slurry flow out of the crystallizer (denoted by *Q* = 0) and neglecting the phenomena of breakage and agglomeration (represented by *B* = *D* = 0), the population balance equation (PBE) can be simplified as follows:

(4) $$\frac{\partial n}{\partial t}+\frac{\partial \left(Gn\right)}{\partial L}=0$$

#### 3.2.2. Nucleation and Growth

The nucleation rate of crystals is predominantly governed by the secondary nucleation property of the crystalline material as well as the fluid flow characteristics of the solution present within the crystallizer. Botsaries have previously alluded to the fact that the growth properties of nuclei produced through the collision process appear to diverge significantly from those of larger crystals [12]. The calculations involved in determining the *B_S_* were based on well-established theoretical frameworks.

(5) $$B_{s}=E_{t}F_{1}F_{2}$$

Here, *B_S_* represents the nucleus formation rate, which arises from the secondary nucleation in the corresponding vessel. The nucleation process can be divided into three distinct stages: the overall energy transport, represented by the energy transport efficiency (*E_t_*); the aggregation breakup, determined by the collision efficiency (*F*_1_); and the formation of nuclei, specified by specific crystal fragmentation constants (*F*_2_). According to Sung’s hypothesis, the observed number of nuclei strongly depends on the solution supersaturation and magnitude of fluid shear forces [13].

Given the incomplete understanding of the nucleation mechanism, it is not possible to express these two functions using mathematical equations. The inclusion of supersaturation or fluid shear forces is recommended for establishing a more comprehensive model. Based on the Botsaries’ model, the nucleation rate was determined using the following equation [14]:

(6) $$B_{s}=K_{b}f(Geom)M_{T}{}^{i}S^{j}N_{P}{}^{k}$$

where *K_b_* is a constant nucleation rate. The exponent in this equation represents the nucleation order. These empirically fitted coefficients reflect the degrees of freedom of the model presented in this paper. *S* represents the supersaturation ratio, and *M_T_* signifies the slurry density. The secondary nucleation rate constant is expressed as follows [15]:

(7) $$K_{b}=k_{0}\exp(-E/RT)$$

where *k*_0_ signifies the pre-exponential factor, *E* denotes the corresponding activation energy, *R* symbolizes the universal gas constant, and *f(Geom)* is a function of the geometry of the crystallizer. To simplify the expression, a parameter *λ* was introduced to account for the similarities in the geometry and size of the crystallizer.

(8) $$B_{s}\propto K_{b}\lambda^{d}M_{T}{}^{i}S^{j}N_{P}{}^{k}$$

The nucleation rate can be evaluated as a functional relationship among the solution supersaturation ratio (*S*), suspension density (*M_T_*), and fluid shear force (*N*) based on the correlation reported by Barrett [16]:

(9) $$B_{s}=K_{b}M_{T}{}^{i}S^{j}N_{P}{}^{k}$$

The assembly nucleation rate was estimated by Giulietti as the sum of the primary and secondary nucleation rates [17]. (This paper demonstrates that the fluid shear force is expressed by the stirring rate):

(10) $$B_{0}=B_{s}+B_{P}=K_{b}M_{T}{}^{i}S^{j}N_{P}{}^{k}$$

The secondary nucleation of crystals is induced by the presence of crystal seeds, and the nucleation rate can be expressed using the simple power-law model proposed by Botsaris et al. [11]:

(11) $$B_{0}=k_{0}\exp\left(-\frac{E}{RT}\right)M_{T}{}^{i}S^{j}N_{P}{}^{k}$$

where *i*, *j*, and *k* represent the nucleation orders. If the same type of crystalline material is suspended within a supersaturated solution, crystals with similar morphologies will exhibit uniform growth rates. For instance, if a crystal exhibits a linear dimension of Δ*L* when allowed to grow within a supersaturated solution, it will consistently exhibit similar linear dimensions throughout the growth process. This phenomenon indicates that the growth rate of the crystal is independent of the initial particle size, which is consistent with theΔ*L* law. Consequently, if the growth process of a crystal conforms to the Δ*L* law, it can be classified as exhibiting a particle–size–independent growth. Empirical equations are commonly employed to calculate the rate of crystal growth:

(12) $$G=k_{G}\exp\left(-\frac{E}{RT}\right)S^{m}N_{P}{}^{n}$$

where *m* and *n* represent the growth kinetics exponents. To simplify the particle beam method solution, this study introduces a transient transformation method to track the size distribution of crystals by solving the matrices (*m_j_*) [18,19]:

(13) $$m_{j}=\int_{0}^{\infty }n\cdot L^{j}dL,\begin{array}{cc} & j=0,1,2,3\ldots \end{array}$$

The temporal derivative of the left-hand side of the above equation is derived as follows:

(14) $$\frac{dm_{0}}{dt}=\frac{d\int_{0}^{\infty }ndL}{dt}=\frac{dN}{dt}=B_{0}$$

(15) $$\frac{dm_{1}}{dt}=\frac{d\int_{0}^{\infty }nLdL}{dt}=\frac{d\lambda_{T}}{dt}=m_{0}G$$

#### 3.2.3. Nucleation Theory

According to the classical nucleation theory, the homogeneous nucleation rate can be expressed as follows:

(16) $$J=A\exp\left(-\frac{\Delta G^{*}}{kT}\right)$$

where *J* refers to the number of nuclei that attain the critical size per unit volume per unit time, ΔG* signifies the free energy required to form a critical nucleus, *k* represents the Boltzmann constant, and T signifies the temperature. The crystallization process results in the transformation of the Gibbs free energy (Δ*G*). It is a well–established concept in the field of physical chemistry [20,21,22,23]. If we assume that the nuclei generated through cluster aggregation have a spherical shape, then the expression for the critical free energy takes the following form:

(17) $$\Delta G=4\pi {\left(r^{*}\right)}^{2}\gamma +\frac{4}{3}\pi {\left(r^{*}\right)}^{3}\Delta G_{V}$$

where *γ* represents the interfacial energy in J/m^2^, and r* represents the critical nucleus radius in meters. When the clusters converge into a critical crystal nucleus, the critical nucleation radius can be obtained by differentiating with respect to r in Equation (17):

(18) $$\frac{d\left(\Delta G\right)}{dr}=8\pi r^{*}\gamma +4\pi {\left(r^{*}\right)}^{2}\Delta G_{V}=0\Rightarrow r^{*}=-\frac{2\gamma }{\Delta G_{V}}$$

By substituting Equation (18) into Equation (17), the critical free energy of the nucleus can be expressed as follows:

(19) $$\Delta G^{*}=\frac{4}{3}\pi {\left(r^{*}\right)}^{2}\gamma$$

Equation (20) is transformed from the Gibbs-Thomson equation as follows:

(20) $$\ln S=\ln\frac{C}{C^{*}}=\frac{2\gamma V_{m}}{kTr^{*}}$$

where C* represents the equilibrium saturation of the solution at the operating temperature, expressed in grams per milliliter. Furthermore, *C* refers to the actual concentration of the solution in grams per milliliter, whereas *V_m_* signifies the molar volume of the crystal, expressed in cubic meters per mole. The saturation of potassium sulfate in water was calculated using the following empirical formula:

(21) $$\begin{array}{l} C^{*}=7.1431+9.45\times 10^{-2}T+2.8\times 10^{-3}T^{2}-5.0\times 10^{-5}T^{3}\\ +4.0\times 10^{-7}T^{4}-1.0\times 10^{-9}T^{5} \end{array}$$

The classical primary homogeneous nucleation theory suggests that solute molecules in supersaturated solutions combine with one another to form crystalline embryos. The Gibbs free energy of the crystalline embryo is:

(22) $$\Delta G^{*}=-\left(\frac{k_{v}r^{3}}{\nu }\right)kT\ln S+k_{a}r^{2}\gamma$$

The variables *k_v_* and *k_a_* represent the respective volume and surface area shape factors. *ν* represents the molecular volume.

In a supersaturated solution characterized by homogeneity, as the number of solute molecules present in the crystal embryo increased, a transient increase in Gibbs free energy (ΔG*) was initially observed. The maximum value of ΔG* corresponds to the nucleation potential barrier, which represents the minimum energy required for the formation of a critical crystal nucleus. The corresponding critical crystal embryo size was designated as r*. Crystals smaller than r* exhibit a gradual melting process, whereas those exceeding r* undergo gradual growth and eventually transform into crystal nuclei. By setting $\frac{\partial \Delta G}{\partial r}=0$ the critical nucleation size r*, the requisite critical nucleation potential barrier required for primary nucleation in a homogeneous solution can be determined. The critical molecular number, *i*, can be used to evaluate the spontaneity of primary nucleation [24]:

(23) $$r^{*}=\frac{2k_{a}V_{m}\gamma }{3k_{v}kT\ln S}$$

(24) $$\Delta G=\frac{k_{a}{\left(r^{*}\right)}^{2}\gamma }{3}=\frac{4k_{{}_{a}}^{2}V_{m}{}^{2}\gamma^{2}}{27k_{{}_{v}}^{2}k^{2}T^{2}\ln^{2}S}$$

(25) i*=kv(r*)3ν=(2kaVm23γ3kv23kTlnS)3

The classical primary homogeneous nucleation rate *J* can be expressed in the Arrhenius form

(26) $$J=A\exp\left(-\frac{4k_{{}_{a}}^{2}V_{m}{}^{2}\gamma^{2}}{27k_{{}_{v}}^{2}k^{2}T^{2}\ln^{2}S}\right)$$

By leveraging the logarithmic transformation, a correlation can be discerned between the induction period and supersaturation ratio.

(27) $$\ln t_{ind}=K+\frac{4k_{{}_{a}}^{2}V_{m}{}^{2}\gamma^{2}}{27k_{{}_{v}}^{2}k^{2}T^{2}\ln^{2}S}$$

The relationship between ln*t_ind_* and 1(lnS)2 at should demonstrate a linear pattern at constant temperature, with a slope of *b*:

(28) $$b=\frac{4k_{{}_{a}}^{2}V_{m}{}^{2}\gamma^{2}}{27k_{{}_{v}}^{2}k^{2}T^{2}}$$

The surface entropy factor *f* plays a significant role in determining the level of surface roughness exhibited by a crystalline material. It is widely acknowledged that an increase in the magnitude of *f* signifies a smoother surface morphology and, subsequently, a more intricate growth process. According to the insightful observations of David, the surface entropy factor can be computed using the solid–liquid interfacial free energy data [25]:

(29) $$f=\frac{4V^{\frac{2}{3}}\gamma }{kT}$$

The value of the surface entropy factor of a crystal is crucial for determining its growth mechanism and polymorphic/morphological properties of the crystals. When the surface entropy factor of a crystal falls below 3, it suggests that the crystal surface exhibits a relatively rough texture, enabling continuous growth. In contrast, when the surface entropy factor exceeded 5, the crystal surface became smooth and experienced relatively slow growth, predominantly through spiral growth or spiral dislocation growth. However, when the value of f lies between 3 and 5, the crystal surface presents a relatively smooth texture, making secondary nucleation possible. This parameter holds significance for a deeper understanding of the crystal growth mechanism and for enhanced control over the polymorphism and morphology of crystals [26,27].

## 4. Conclusions

Firstly, the effects of the supersaturation level and temperature upon the nucleation kinetics of K_2_SO_4_ were explored by measuring of the initiation period. Subsequently, the crystallization kinetic information of K_2_SO_4_ was obtained using the batch dynamic methodology of sequential sampling. The nucleation rates and growth dynamics were derived by fitting of these acquired data. The rectangular block–shaped size increased with the supersaturation ratio and temperature (20–120 μm). Higher temperatures and supersaturation favor larger crystal growth decreases with increasing supersaturation ratio and temperature. The temperature impact is significant above a ratio of 1.22, whereas supersaturation changes are critical at 1.20 for nucleation. Homogeneous nucleation occurred at high supersaturations (above 1.22), and heterogeneous nucleation occurred at lower ratios (below 1.20). The increased temperature and supersaturation lower the critical free energy (ΔG*), radius (r*), and number (i*) for nucleation, thereby increasing the nucleation rate (J*). The rectangular shape remained unaffected by temperature and supersaturation changes. The size increased with supersaturation and temperature. Based on the particle density distribution, the supersaturation ratio and temperature positively influenced the growth.

## Figures and Tables

**Table 1:** The coefficients for the crystallization kinetics expression.

Coefficients	Nucleation Rate (*B*_0_)	Growth Rate (*G*)
*k_B_*	7.344 × 10^6^	NS
*k_G_*	NS	3.233 × 10^−6^
*E*	1.4555 × 10^4^	5.433 × 10^3^
*i*	0.3445	NS
*j*	1.6522	NS
*k*	1.9876	NS
*m*	NS	0.4655
*n*	NS	0.7455
*R* ^2^	0.989	0.978

## Data Availability

All data generated or analyzed during this study are included in this manuscript. The data included in this study are available upon request from the corresponding author.
